# Risk factors and complications associated with intra-operative or post-operative identification of a PFO in cardiac surgery patients: A cohort study

**DOI:** 10.3389/fneur.2022.1057479

**Published:** 2023-01-10

**Authors:** Driss Laghlam, Lucas Coroyer, Paul-Jun Martial, Philippe Estagnasie, Pierre Squara, Lee S. Nguyen

**Affiliations:** Department of Cardiology and Critical Care, Clinique Ambroise Paré, Neuilly-sur-Seine, France

**Keywords:** patent foramen ovale, cardiac surgery, stroke, patent foramen ovale closure, ascending aorta surgery, mitral valve surgery

## Abstract

**Introduction:**

It is unknown whether patent foramen ovale (PFO) reopening in the peri-operative setting of cardiac surgery affects the risk for stroke and post-operative outcomes.

**Methods:**

We performed a single-center, retrospective study based on a prospectively collected database in a tertiary cardiac surgery center. Using logistic regression, we assessed risk factors of PFO finding around surgery and subsequent clinical complications.

**Results:**

Between January 2007 and July 2019, 11034 patients who underwent cardiac surgery in our center were included. A total of 233 patients (2.1%) presented a finding of PFO including 138 per-operative disclosures and 95 post-operative finding for hypoxemia. In the whole cohort, the mean age was 68.4 ± 11.5 years including 73.9% of men. Post-operative PFO finding was associated with more ischemic strokes compared with per-operative finding and control group [7(7.4%) vs. 3(2.2%) vs. 236(2.2), respectively; *p* = 0.003]. Moreover, patients with post-operative PFO reopening experienced a higher rate of pneumonia, reintubation, and longer length of stay in the ICU. Post-operative reopening of PFO, but not per-operative finding, was independently associated with ischemic strokes {adjusted odds-ratio = 3.5, 95% confidence interval (CI) [1.6–7.8]; *p* = 0.002}. Other variables associated with stroke incidence included age, mitral valve surgery, and ascending aorta surgery. Per- or post-operative PFO closure was associated with reduced adverse respiratory outcomes and a trend of the lower cerebral ischemic event.

**Conclusion:**

Patent foramen ovale finding incidence in peri-operative cardiac surgery care was rare (2%) but post-operative finding of PFO was associated with a increased risk of ischemic strokes, worsened respiratory outcomes, and prolonged hospitalization.

## Introduction

Patent foramen ovale (PFO) is a frequent pathology with an incidence of 25% in the general population of healthy subjects (1). Its reopening during peri-operative care around cardiac surgery may have severe consequences such as hypoxemia and ischemic events, including strokes. Its management from means of diagnosis to treatment remains ambiguous for now.

Patent foramen ovale is associated with a marked increase in cryptogenic strokes, due to paradoxical embolism (2–4). Recent randomized trials have been published showing a significant decrease in the risk of stroke recurrence after PFO closure among patients with cryptogenic stroke (5–8). This risk also exists in patients undergoing non-cardiac surgery. It is probably increased by peri-operative venous stasis and hemodynamic changes that increase right-side pressures, such as orotracheal intubation and discontinuation of anticoagulants, making patients with a PFO a subgroup at high risk of ischemia during the peri-operative period (9, 10).

Multiple conditions can lead to PFO reopening, most of them involving modifications of the thoracic geometry, an increase of the right pressure or modifications of the venous return flow toward the inter-atrial septum (11), COPD (12), obstructive sleep apnea (13), positive end-expiratory pressure under mechanical ventilation (14), dilatation of the ascending aortic root (15, 16), major thoracic surgery (17, 18), and diaphragmatic paralysis (19).

Hence, patients undergoing cardiac surgery seem more likely exposed to PFO reopening because of sternotomy, pericardiotomy, possible ascendant aortic interventions, frequent diaphragmatic paralysis, and orotracheal intubation with mechanical ventilation and PEEP (14, 20).

We aimed to describe and identify risk factors of PFO reopening and to study the relation between PFO reopening and post-operative complications.

## Methods

We conducted a single-center retrospective cohort study, from a prospectively collected database. We included all patients aged more than 18 years who underwent cardiac surgery with cardiopulmonary bypass, from June 2007 to July 2019. Patients who died during surgery were not included in the analysis, considering that the main goal was to explore post-operative outcomes. All data are part of the Registry for the Improvement of Post-operative OutcomeS in Cardiac and Thoracic Surgery (RIPOSTE) database (https://clinicaltrials.gov/ct2/show/NCT03209674) (21–24).

This registry systematically collected pre-operative variables of patients, allowing them to compute risk scores, and also collected surgical procedure information (including the type of procedure and circulatory bypass information) and post-operative clinical outcomes with a follow-up extended until discharge from the hospital. According to the French law, consent to participate was waived but patients' opposition to the use of anonymized data was systematically sought. Data were anonymized as per national regulation and used with the approval of an institutional review board committee.

### Surgical and peri-operative management

Peri-operative management was standardized. All patients had the right internal jugular central venous catheter. All procedures involved a full- or mini-sternotomy approach. Mammary arteries and saphenous veins were used as coronary bypass grafts. Myocardial protection was provided using a normothermic continuous blood cardioplegia solution. A transthoracic echocardiography was systematically performed in all patients undergoing cardiac intervention but without systematic research on PFO. A transesophageal echocardiography was systematically performed in the operative room for mitral and tricuspid surgery and if needed for other procedures. During surgery (except for the cross-clamping period), mechanical ventilation was set at a tidal volume of 6 ml/kg predicted body weight and a positive end-expiratory pressure level of 5 cm H_2_O, the respiratory rate was adjusted to maintain normocapnia, and the inspired fraction of oxygen (FiO_2_) was set to keep PaO_2_ below 100 mmHg. In the intensive care unit (ICU), standard ventilation protocol included lung-protective with tidal volume of 6 mL/kg ideal body weight, PEEP of 5 cm H_2_O, FiO_2_ set to obtain PaO_2_ above 100 mmHg, and inspiration/expiration time ratio = 1:2. However, the adaptation of respiratory settings and the administration of other therapeutics were left to the appreciation of the clinicians. After the surgery, the patients were transferred to the ICU department, where an assessment of the absence of bleeding, respiratory and hemodynamic stability, and normothermia was performed before discontinuing sedatives and performing a spontaneous breathing trial.

In our center, standard medical treatment for post-operative PFO reopening included lowering right cavity pressure by minimizing fillings or even diuretics, as well as lowering end-positive expiratory pressure during mechanical or non-invasive ventilation, if necessary, due to the effects on intrathoracic pressures. In cases of refractory hypoxemia that did not improve spontaneously, the percutaneous closure procedure was quickly considered.

### Outcome definitions

The main objective was to explore risk factors and outcomes of patients who presented a finding of PFO during or after the index surgery compared with those who did not. Outcomes were assessed during the hospital stay.

Patent foramen ovale reopening was defined using ultrasound diagnostic criterion in transthoracic or transesophageal echocardiography using contrast perfusion, that is, early appearance of microbubbles between the right and left atrium. We evaluated the presence of PFO by transesophageal echocardiography with contrast injection in immediate pre-operative settings in the operative room or by transthoracic echocardiography with contrast +/– Valsalva maneuver in post-operative non-intubated patients.

Stroke was defined by the appearance of a central neurological deficit associated with cerebral imaging compatible with an ischemic process, while the transient ischemic attack was not associated with abnormalities in cerebral imaging. The local practice was to perform an MRI as a first-line imaging modality in acute ischemic stroke suspicion when available and in the absence of contraindication; and failing that, a head CT. Post-operative atrial fibrillation was defined as at least one episode of atrial fibrillation after surgery, regardless of its duration. Post-operative pneumonia was defined based on a new lung infiltrate on daily chest X-ray associated with at least two of the following finding: temperature ≥38.3 or < 36°C, white blood cell count >12,000 cells/mm^3^ or < 5,000 cells/mm^3^, and purulent secretion.

### Statistical analysis

Continuous variable distribution was assessed using the Shapiro–Wilk test. They were expressed as mean ± standard deviation when satisfying normal distribution, and median [interquartile range] otherwise. Categorical variables were expressed as numbers (percentages). The Chi-2 or Fisher exact tests were used to compare categorical variables. Comparisons of continuous variables were performed by two-way ANOVA for repeated measurements followed by paired Student t-tests with Bonferroni correction if needed, or by Friedman test followed by Wilcoxon tests if needed, according to data distribution.

To assess the association between PFO reopening and ischemic strokes, we used multivariable logistic regression models constructed with a stepwise mixed method with a *p*-value entry threshold of 0.15 and an exit threshold of 0.1. All variables subsequently identified were considered clinically relevant, and none was rejected. Linearity was checked. A two-sided *p*-value of < 0.05 was considered statistically significant. Statistical analysis was performed with SPSS 25.0 (IBM, Armonk, United States of America).

## Results

During the study period (from June 2007 to July 2019), 11,089 patients underwent cardiac surgery procedures with cardiopulmonary bypass in our center. Among them, we included 11,034 patients in the final analysis (55 patients were excluded: 21 dying during surgery and 34 with missing pre- or/and post-operative data which did not allow further analyses, see Flow-Chart in Supplementary Figure 1). We have separated the cohort into three distinct groups: a control group (no PFO found), a group with intraoperative discovery, and a third group with the post-operative finding of PFO. A total of 233 patients (2.1%) presented a finding of PFO in the peri-operative setting. Among them, 138 were per-operative and 95 post-operative finding.

The baseline and per-operative characteristics of the whole cohort are shown in Table 1. Overall, the mean age was 68.4 ± 11.5 years including 73.9% of men. Compared with the control and post-operative finding of the PFO group, patients with per-operative finding of PFO were younger, with fewer diabetic [2,777 (26.0%) vs. 19 (20%) vs. 14 (10.1%), respectively, *p* < 0.0001], lower body mass index (26.6 ± 4.5 vs. 27.0 ± 4.7 vs. 24.7 ± 4.0, respectively; < 0.0001), higher rate of pre-operative atrial fibrillation [913 (8.5%%) vs. 12 (12.6%) vs. 35 (25.4%), respectively; *p* < 0.0001], and higher pre-operative pulmonary artery systolic pressure above 35 mmHg [2,243 (29.5%) vs. 28 (29.5%) vs. 50 (36.2%), respectively; *p* < 0.0001]. The per-operative finding of PFO occurred more frequently after mitral valve procedures as well as after tricuspid valve surgery. Conversely, the post-operative finding of PFO occurred more frequently after ascending aorta surgery.

**Table 1:** Baseline and pre-operative characteristics.

	**Control group (*n =* 10,801)**	**Per-operative PFO finding (*n =* 138)**	**Post-operative PFO finding (*n =* 95)**	***p***
**Baseline characteristics**				
Age, mean ± SD	68.4 ±11.5	65.6 ± 13.2	67.1 ± 10.8	0.02
Male sex, *n* (%)	7,993 (74)	91 (65.9)	75 (78.9)	0.053
Smoker status, *n* (%)	3,327 (31.1)	33 (23.9)	37 (38.9)	0.049
Hypertension, *n* (%)	5,738 (53.7)	55 (39.9)	58 (61.1)	0.004
Diabetes, *n* (%)	2,777 (26.0)	14 (10.1)	19 (20)	< 0.0001
Body mass index (kg/m^2^)	26.6 ± 4.5	24.7 ± 4.0	27.0 ± 4.7	< 0.0001
COPD, *n* (%)	692 (6.5)	10 (7.2)	6 (6.3)	0.92
Left ventricular ejection function ≥50%, *n* (%)	8,958 (82.9)	124 (89.9)	79 (83.1)	0.10
Left ventricular ejection function 31–49%, *n* (%)	1,578 (14.6)	10 (7.2)	13 (13.7)	0.049
Left ventricular ejection function ≤ 30%, *n* (%)	265 (2.5)	4 (2.9)	3 (3.2)	0.83
Peripherical arterial disease, *n* (%)	1,212 (11.2)	8 (5.8)	17 (17.9)	0.02
Pulmonary artery systolic pressure >35 mmHg, *n* (%)	2,243 (29.5)	50 (36.2)	28 (29.5)	< 0.0001
Pulmonary artery systolic pressure >55mmHg, *n* (%)	409 (3.8)	15 (15.9)	1 (1.1)	< 0.0001
Previous atrial fibrillation, *n* (%)	913 (8.5)	35 (25.4)	12 (12.6)	< 0.0001
**Per-operative characteristics**				
Coronary bypass grafting, *n* (%)	6,617 (61.3)	16 (11.6)	64 (67.4)	< 0.0001
Mitral valve replacement, *n* (%)	731 (6.8)	48 (34.8)	3 (3.2)	< 0.0001
Mitral valve repair, *n* (%)	814 (6.8)	45 (32.6)	3 (3.2)	< 0.0001
Aortic valve surgery, *n* (%)	3,701 (34.3)	15 (10.9)	22 (23.2)	< 0.0001
Ascending aorta surgery, *n* (%)	758 (7.0)	5 (3.6)	18 (18.9)	< 0.0001
Tricuspid valve surgery *n* (%)	327 (3.0)	53 (38.4)	1 (1.1)	< 0.0001
Urgent surgery, *n* (%)	630 (5.8)	5 (3.6)	7 (7.4)	0.43
Redux	654 (6.1)	15 (10.9)	4 (4.2)	0.047

Post-operative outcomes of the three groups are shown in Table 2. Post-operative PFO finding was associated with more ischemic strokes compared with per-operative finding and control group [7 (7.4%) vs. 3 (2.2%) vs. 236 (2.2), respectively; *p* = 0.003]. Moreover, patients with post-operative finding PFO reopening experienced a higher rate of pneumonia, reintubation, and longer length of stay in the ICU. Patients with per-operative finding of PFO had a higher rate of post-operative atrial fibrillation and vasopressor use. In-hospital mortality was 4.2% in the whole cohort and did not differ between the three groups.

**Table 2:** Post-operative outcomes.

	**Control group (*n =* 10,801)**	**Per-operative PFO finding (*n =* 138)**	**Post-operative PFO finding (*n =* 95)**	***p***
Pneumonia, *n* (%)	989 (9.2)	15 (10.9)	27 (28.4)	< 0.0001
Reintubation, *n* (%)	376 (3.5)	6 (4.3)	13 (13.7)	< 0.0001
Duration of mechanical Ventilation(hours),median[IQR]	6 [4–8]	6 [4–9]	5 [4–148]	0.33
Cerebral ischemic event, *n* (%)	236 (2.2)	3 (2.2)	7 (7.4)	0.003
Comitial event, *n* (%)	63 (0.6)	3 (2.2)	2 (2.1)	0.01
Diaphragmatic paralysis, *n* (%)	340 (3.1)	3 (2.2)	12 (12.6)	< 0.0001
New onset of postoperative atrial fibrillation, *n* (%)	2,598 (24.0)	67 (48.6)	20 (21.1)	< 0.0001
Vasopressors use, *n* (%)	1,435 (13.3)	40 (29.0)	15 (15.8)	< 0.0001
Mediastinitis, *n* (%)	229 (2.3)	0 (0)	2 (2.1)	0.22
Length of stay in ICU, days median [IQR]	4 [3–5]	4 [3–5]	6 [4–8.5]	< 0.0001
Length of stay in hospital, days, median [IQR]	13 [11–16]	14 [12–17.8]	16 [13–23]	< 0.0001
In-hospital mortality, *n* (%)	446 (4.1)	4 (2.9)	8 (8.4)	0.09

### Risk factors of PFO reopening

**Figure 1:**
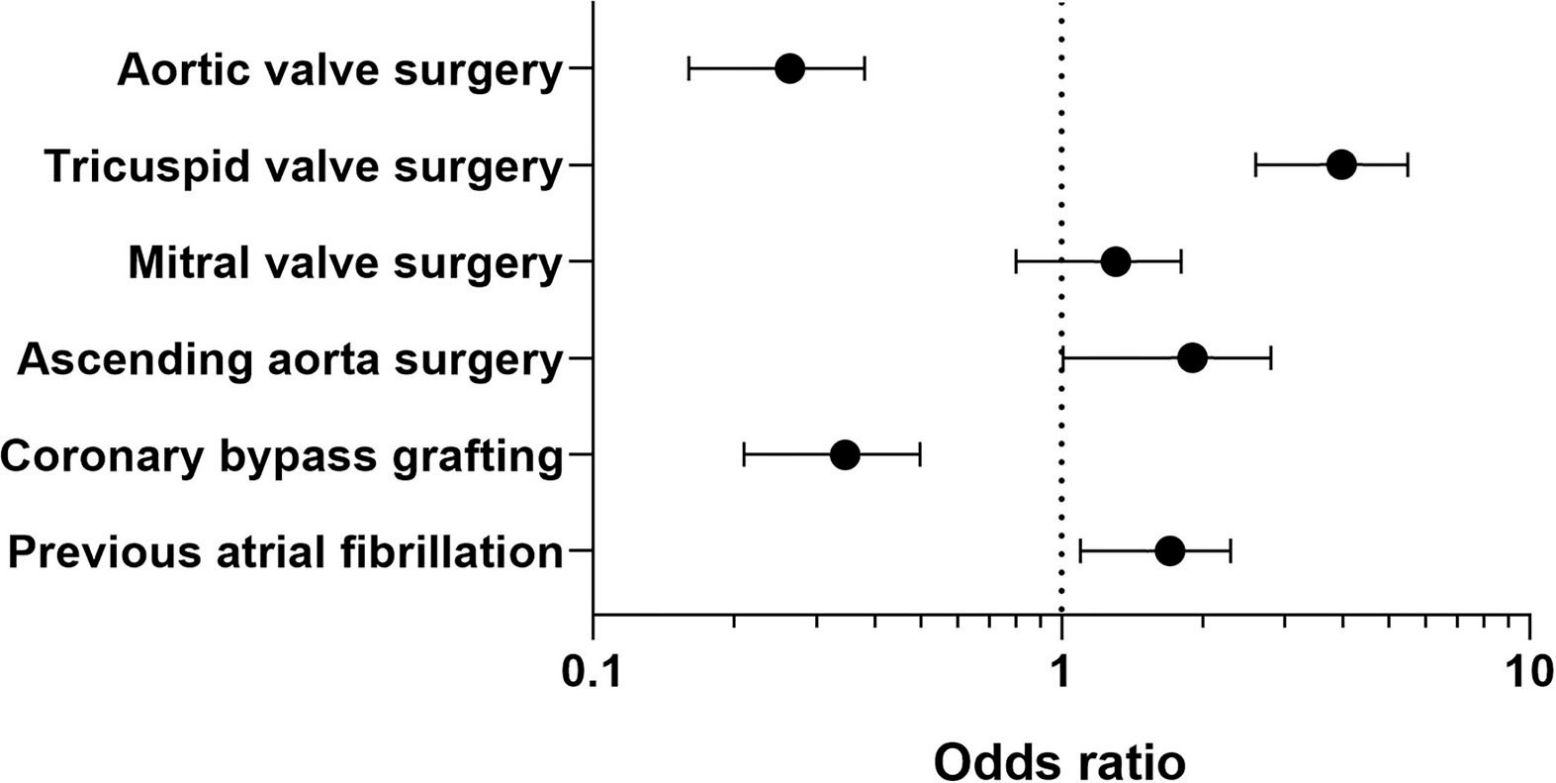
Predictors of PFO finding in multivariate analysis.

Variables associated with PFO reopening are shown in Supplementary Table 1. After adjusting for confounding variables by using multivariable logistic regression, variables independently associated with PFO reopening were: pre-operative atrial fibrillation [adjOR = 1.6, 95%CI (1.1–2.3), *p* = 0.01], CABG [adjOR = 0.32, 95%CI (0.21–0.50), *p* = 0.001], aortic valve procedure [adjOR = 0.25, 95%CI (0.16–0.38), *p* = 0.0001], combined ascending aorta procedures [adjOR = 1.7 95%CI (1.01–2.80), *p* = 0.048], and tricuspid valve surgery [adjOR = 3.7 95%CI (2.6–5.5), *p* = 0.0001] (see Figure 1).

### Risk factors of post-operative ischemic strokes

**Figure 2:**
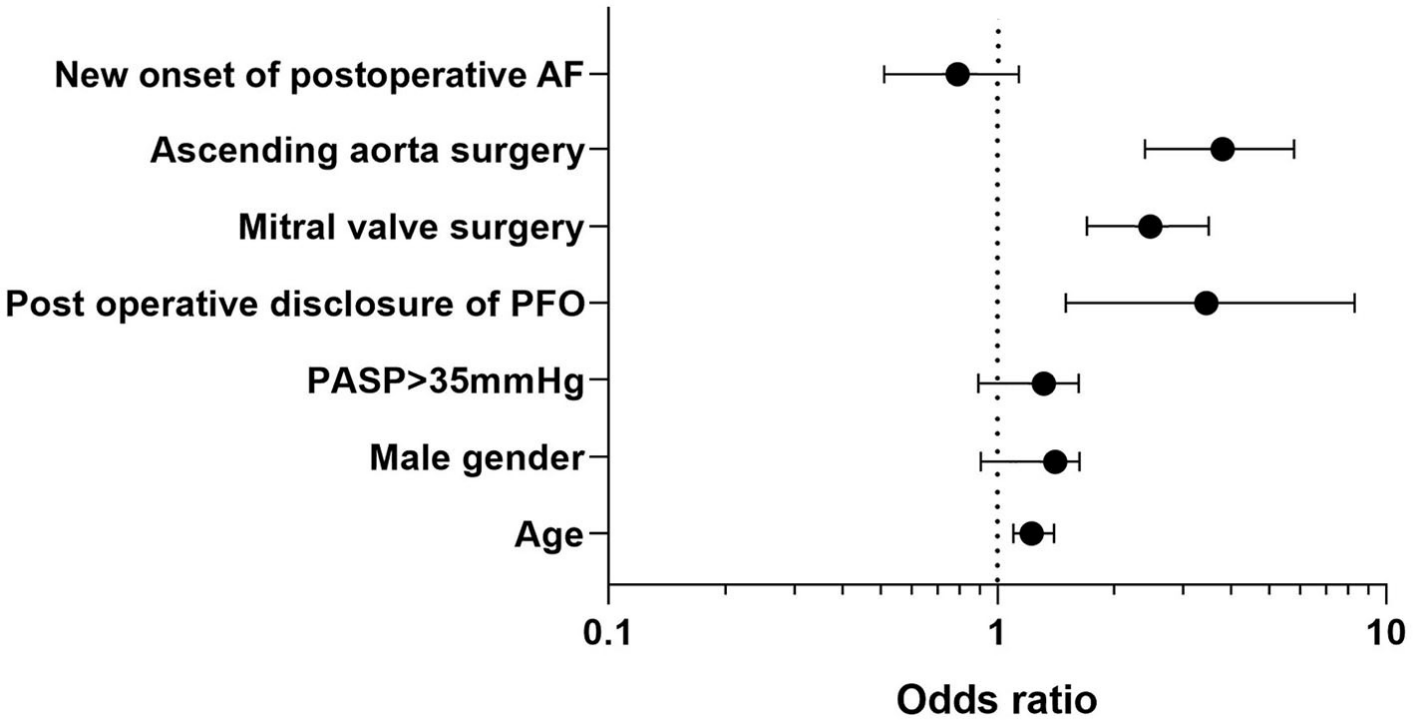
Variables associated with ischemic strokes in multivariable logistic regression analysis.

Variables associated with post-operative stroke are shown in Supplementary Table 2. After adjusting for confounding variables by using multivariable logistic regression, variables independently associated with post-operative stroke were post-operative PFO reopening [adjOR = 3.5 (1.6–7.8), *p* = 0.005], age [per-1-year increase, adjOR = 1.02 (1.01–1.04), *p* = 0.0001], mitral valve procedures [adjOR = 2.4 (1.8–3.3), *p* = 0.0001], and combined ascending aorta procedures [adjOR = 3.1 (2.1–4.5), *p* = 0.0001], whereas men [adjOR = 1.2 (0.9–1.5), *p* = 0.26], elevated systolic pulmonary artery pressure above 35 mmHg [adjOR = 1.1 (0.9–1.5), *p* = 0.38], and new onset of post-operative atrial fibrillation [adjOR = 0.8 (0.55–1.15), *p* = 0.23] were not associated with (see Figure 2).

### Effect of PFO closure procedure on clinical outcomes

Among patients with PFO reopening, 151 underwent closures, including all 138 per-operative discoveries who underwent concomitant surgical corrections. Among 95 post-operative diagnoses, 13 underwent percutaneous closure and 82 received non-interventional medical treatment only.

Per- or post-operative closure of PFO was associated with a significantly lower rate of pneumonia [18 (11.9%) vs. 24 (29.3%); *p* = 0.002], lower rate of reintubation [8 (5.2%) vs. 11 (13.4%); *p* = 0.03], and reduced length of stay in ICU 4 [3–5] days vs. 5.5 [4–8] days; *p* < 0.001) (see Supplementary Table 3). There was a trend of a higher rate of cerebral ischemic events and in-hospital mortality in patients without the closure of PFO without significant difference [6 (7.3%) vs. 4 (2.6%); *p* = 0.17 and 7 (8.5%) vs. 5 (3.3%); *p* = 0.08, respectively].

## Discussion

This study yielded three main finding: (1) PFO finding in peri-operative cardiac surgery care was a rare event (~2%), (2) post-operative PFO reopening was associated with a increased risk of ischemic strokes, worsened respiratory outcomes, and prolonged hospitalization, and (3) PFO closure was associated with reduced adverse respiratory outcomes and a trend toward a lower cerebral ischemic event.

We reported a large cohort study describing the consequences of PFO finding in the peri-operative period of patients undergoing cardiac surgery. There is no consensus about pre-operative screening and closing of PFO. Some teams have proposed to close PFO during cardiac surgery because of the ease and the rapidity of the surgical procedure. However, this procedure remains uncommon. A 2005 national survey in the United States estimated that 27% of cardiac surgeons routinely closed an intraoperatively diagnosed PFO (25). In clinical practice, PFO closure is performed when it does not significantly extend the operative time or when the procedure does not entail additional morbidity (i.e., procedures involving an atriotomy such as mitral or tricuspid valve surgery) (26, 27). A large retrospective study reported that incidental PFO is common in patients undergoing cardiac surgery (17%) but was not associated with increased peri-operative morbidity or mortality. However, surgical closure of PFO appears unrelated to long-term survival and may increase post-operative stroke risk (28).

We found that patients with post-operative PFO finding experienced significantly more cerebral ischemic events after cardiac surgery compared with per-operative PFO finding and control group [7 (7.4%) vs. 3 (2.2%) vs. 236 (2.2), respectively; *p* = 0.003]. Previously, Krasuki et *al*. described in a large cohort study of 13,092 patients that 2,277 patients (17%) presented with intraoperative PFO. In this study, there was no difference in mortality, post-operative stroke, or length of hospital stay between patients with and without PFO. However, in the subgroup of patients who underwent surgical PFO closure [639/2,277 (28%)], there was a increased risk of developing post-operative stroke (2.8 vs. 1.2%, *P* = 0.04; OR = 2.47, 95% CI [1.02–6.00]) (28). Our results are conflicting, as we reported that only a post-operative reopening of PFO, rather than a per-operative finding, was independently associated with stroke. Other risk factors of stroke included the previously mentioned variables: age, male gender, mitral valve surgery, ascending aorta surgery, and pre-operative PASP >35 mmHg. Yet, Krasuki et al. reported data from a large database with systematic research of PFO and with a high rate of per-operative closure (28%). Our study design differs because we reported here only the outcomes of patients with the decision of per-operative closure or with the post-operative finding of PFO but not the incidence and outcomes of pre-operative PFO disclosure.

Patent foramen ovale has already been described as an important risk factor for stroke (7, 8); with a correlation among the size of the PFO, the number of microbubbles during the bubble test (and therefore the importance of the shunt), and the risk of embolism (29). Stroke is a frequent event after cardiac surgery affecting ~1–5% of patients, leading to disability and higher post-operative mortality (30–32). In our study, the post-operative stroke rate was 2.2%, consistent with a recent observational study (2.16% on 10,250 patients) (32), and somewhat higher than the ~1% rate yielded in a meta-analysis based on 174,969 patients (33). Remarkably, other factors associated with stroke after cardiac surgery included age, hypertension, diabetes, duration of cardiopulmonary bypass, type of intervention, and aorta atherosclerosis (30, 31).

Furthermore, PFO is associated with an increased risk of stroke in the peri-operative setting even after non-cardiac surgery (10). In the cardiac surgery setting, post-operative prothrombotic status, central venous catheter, or other intravascular dispositive could potentialize the risk of stroke in case of post-operative PFO reopening. We also reported the same association between PFO and post-operative atrial fibrillation described by Djaiani et al. (34), which could contribute to post-operative risk of stroke in this population, although it does not emerge as an independent factor in our analyses.

We found that tricuspid valve, ascending aorta surgery, and previous atrial fibrillation were independent variables associated with an increased risk for PFO finding. Tricuspid valve surgery and previous atrial fibrillation could be associated with higher pre-operative PASP and right atrium dysfunction due to dilatation, explaining a increased risk of high right atrial pressure and PFO reopening. In addition, we confirm that ascending aorta surgery is associated with post-operative PFO finding as previously described (15, 16, 35). Indeed, dilatation of ascending aorta, which constitutes its main surgical indication, was found to lead to counterclockwise rotation of the heart, horizontalization, and a “loose” appearance of the inter-atrial septum favoring PFO reopening (15). Conversely, valve aortic surgery and coronary bypass grafting were independently associated with a risk decrease in PFO finding. We could hypothesize that the absence of per-operative atriotomy reduced the risk of the PFO finding, and that patients with aortic valve and coronary disease had less previous atrial fibrillation and higher right atrium pressure than patients with tricuspid disease.

Among patients with PFO finding, 95 had post-operative PFO finding revealed by marked hypoxemia. Thirteen (14%) required percutaneous closure because of the persistence of significant hypoxemia despite first-line medical treatment. This procedure is easily performed by interventional cardiologists, with few side effects for patients. The most frequent complications identified in randomized studies of percutaneous closure devices were atrial fibrillation (0.6–4.6%), ischemic strokes (0.4%) due to failure to purge, and pericardial effusions (0.6%), which were sometimes life-threatening and may require drainage (5–8, 36).

We acknowledge that our study has several limitations. First, it was a single-center observational study; these results correspond to a specific experience. Second, the retrospective character of this study leads to inherent biases in this type of study. We could not exclude that some events were misreported during the data collection. We did not report data on the size of the shunt in transthoracic or transesophageal echocardiography, which is important data to interpret the risk of occurrence of ischemic events. In addition, we did not have the details on what type of imaging was performed (MRI or CT) and the ischemic strokes pattern in imaging, which does not allow us to conclude on the size and the severity of these ischemic events. Moreover, despite systematic pre-operative echocardiography, there was no standardized pre- and post-operative screening protocol for PFO, and the presence of pre-operative PFO was not reported in the database, so the incidence of PFO was probably underestimated. Therefore, we only reported PFO found and closed during surgery, as well as PFO revealed in the post-operative setting. Moreover, the screening of PFO mainly in patients with hypoxemic post-operative may have overestimated the association with other causes of hypoxemia (pneumonia and reintubation). Third, another major issue is that we are unaware of what medications our patients were taking before or after surgery. We did not report the use and the effects of stopping or starting treatment as anticoagulation and anti-platelet therapy on the risk of stroke. In patients with cryptogenic stroke, there was no clear-cut difference in efficacy found in trials between anti-platelet and oral anticoagulation therapy with warfarin or novel oral anticoagulants, in the absence of atrial fibrillation (37). Usually, in our center, patients undergoing coronary bypass grafting surgery and aortic valve replacement had at least one anti-platelet agent and prophylactic anticoagulation, while patients undergoing mitral and tricuspid surgery were treated immediately with therapeutic anticoagulation post-operatively. All patients with atrial fibrillation received therapeutic anticoagulation.

Fourth, association in multivariate analysis is not causation, and we cannot fully conclude about the effect of PFO reopening on ischemic stroke risk.

Finally, because all patients diagnosed with PFO reopening during surgery were surgically corrected, we did not have a control group to assess the consequences of per-operative surgical closure of PFO.

## Conclusion

In this large single-center observational study, we found that PFO finding incidence in peri-operative cardiac surgery care was ~2%. The post-operative finding of PFO was associated with a increased risk of ischemic stroke, worsened respiratory outcomes, and prolonged hospitalization. Furthermore, post-operative reopening of PFO was an independent risk factor of stroke with more than a three-fold increase.

## Data availability statement

The raw data supporting the conclusions of this article will be made available by the authors, without undue reservation.

## Ethics statement

The studies involving human participants were reviewed and approved by CMC Ambroise Paré Local Ethics Commitee. The patients/participants provided their written informed consent to participate in this study.

## Author contributions

All authors listed have made a substantial, direct, and intellectual contribution to the work and approved it for publication.

## References

[1] Hagen PT Scholz DG Edwards WD. Incidence and size of patent foramen ovale during the first 10 decades of life: an autopsy study of 965 normal hearts. Mayo Clin Proc. (1984) 59:17–20. 10.1016/S0025-6196(12)60336-X 6694427

[2] Ntaios G Papavasileiou V Milionis H Makaritsis K Manios E Spengos K. Embolic strokes of undetermined source in the Athens stroke registry: a descriptive analysis. Stroke. (2015) 46:176–81. 10.1161/STROKEAHA.114.007240 25378429

[3] Handke M Harloff A Olschewski M Hetzel A Geibel A. Patent foramen ovale and cryptogenic stroke in older patients. N Engl J Med. (2007) 357:2262–8. 10.1056/NEJMoa071422 18046029

[4] Cabanes L Mas JL Cohen A Amarenco P Cabanes PA Oubary P. Atrial septal aneurysm and patent foramen ovale as risk factors for cryptogenic stroke in patients less than 55 years of age. A study using transesophageal echocardiography. Stroke. (1993) 24:1865–73. 10.1161/01.STR.24.12.1865 8248969

[5] Mas JL Derumeaux G Guillon B Massardier E Hosseini H Mechtouff L. Patent Foramen Ovale Closure or Anticoagulation vs. Antiplatelets after stroke. N Engl J Med. (2017) 377:1011–21. 10.1056/NEJMoa1705915 28902593

[6] Saver JL Carroll JD Thaler DE Smalling RW MacDonald LA Marks DS. Long-term outcomes of patent foramen ovale closure or medical therapy after stroke. N Engl J Med. (2017) 377:1022–32. 10.1056/NEJMoa1610057 28902590

[7] Ahmad Y Howard JP Arnold A Shin MS Cook C Petraco R. Patent foramen ovale closure vs. medical therapy for cryptogenic stroke: a meta-analysis of randomized controlled trials. Eur Heart J. (2018) 39:1638–49. 10.1093/eurheartj/ehy121 29590333 PMC5946888

[8] Søndergaard L Kasner SE Rhodes JF Andersen G Iversen HK Nielsen-Kudsk JE. Patent foramen ovale closure or antiplatelet therapy for cryptogenic stroke. N Engl J Med. (2017) 377:1033–42. 10.1056/NEJMoa1707404 28902580

[9] Kasner SE Messe SR. Is patent foramen ovale a risk factor for perioperative stroke? Jama. (2018) 319:446–7. 10.1001/jama.2017.21902 29411017

[10] Ng PY Ng AK Subramaniam B Burns SM Herisson F Timm FP. Association of Preoperatively Diagnosed Patent Foramen Ovale With Perioperative Ischemic Stroke. Jama. (2018) 319:452–62. 10.1001/jama.2017.21899 29411032 PMC5839297

[11] Bancal C Arnoult F Krapf L Bonay M. [Patent foramen ovale and hypoxaemia with or without elevated right heart pressures]. Rev Mal Respir. (2011) 28:967–77. 10.1016/j.rmr.2011.07.001 22099402

[12] Soliman A Shanoudy H Liu J Russell DC Jarmukli NF. Increased prevalence of patent foramen ovale in patients with severe chronic obstructive pulmonary disease. J Am Soc Echocardiogr. (1999) 12:99–105. 10.1016/S0894-7317(99)70121-5 9950968

[13] Shanoudy H Soliman A Raggi P Liu JW Russell DC Jarmukli NF. Prevalence of patent foramen ovale and its contribution to hypoxemia in patients with obstructive sleep apnea. Chest. (1998) 113:91–6. 10.1378/chest.113.1.91 9440574

[14] Cujec B Polasek P Mayers I Johnson D. Positive end-expiratory pressure increases the right-to-left shunt in mechanically ventilated patients with patent foramen ovale. Ann Intern Med. (1993) 119:887–94. 10.7326/0003-4819-119-9-199311010-00004 8215000

[15] Eicher JC Bonniaud P Baudouin N Petit A Bertaux G Donal E. Hypoxaemia associated with an enlarged aortic root: a new syndrome? Heart. (2005) 91:1030–5. 10.1136/hrt.2003.027839 15761046 PMC1769048

[16] Shiraishi Y Hakuno D Isoda K Miyazaki K Adachi T. Platypnea-orthodeoxia syndrome due to PFO and aortic dilation. JACC Cardiovasc Imaging. (2012) 5:570–1. 10.1016/j.jcmg.2012.01.015 22595167

[17] Ng SY Sugarbaker DJ Frendl G. Interatrial shunting after major thoracic surgery: a rare but clinically significant event. Ann Thorac Surg. (2012) 93:1647–51. 10.1016/j.athoracsur.2012.02.029 22541195

[18] Marini C Miniati M Ambrosino N Formichi B Tonelli L Di Ricco G. Dyspnoea and hypoxaemia after lung surgery: the role of interatrial right-to-left shunt. Eur Respir J. (2006) 28:174–81. 10.1183/09031936.06.00006405 16816347

[19] Ghani AR Hamid M Dhillon P Ullah W. Tilting the balance: hemidiaphragm paralysis leading to right to left cardiac shunt. BMJ Case Rep. (2018) 11:bcr-2018. 10.1136/bcr-2018-227944 30567185 PMC6301587

[20] Akhter M Lajos TZ. Pitfalls of undetected patent foramen ovale in off-pump cases. Ann Thorac Surg. (1999) 67:546–8. 10.1016/S0003-4975(98)01246-6 10197692

[21] Levy D Laghlam D Estagnasie P Brusset A Squara P Nguyen LS. Post-operative right ventricular failure after cardiac surgery: a cohort study. Front Cardiovasc Med. (2021) 8:667328. 10.3389/fcvm.2021.667328 34195233 PMC8236513

[22] Laghlam D Lê MP Srour A Monsonego R Estagnasié P Brusset A. Diaphragm dysfunction after cardiac surgery: reappraisal. J Cardiothorac Vasc Anesth. (2021) 35:3241–7. 10.1053/j.jvca.2021.02.023 33736912

[23] Taleb Bendiab T Brusset A Estagnasié P Squara P Nguyen LS. Performance of EuroSCORE II and Society of Thoracic Surgeons risk scores in elderly patients undergoing aortic valve replacement surgery. Arch Cardiovasc Dis. (2021) 114:474–81. 10.1016/j.acvd.2020.12.004 33558164

[24] Nguyen LS Baudinaud P Brusset A Nicot F Pechmajou L Salem JE. Heart failure with preserved ejection fraction as an independent risk factor of mortality after cardiothoracic surgery. J Thorac Cardiovasc Surg. (2018) 156:188–93.E2. 10.1016/j.jtcvs.2018.02.011 29530566

[25] Sukernik MR Goswami S Frumento RJ Oz MC Bennett-Guerrero E. National survey regarding the management of an intraoperatively diagnosed patent foramen ovale during coronary artery bypass graft surgery. J Cardiothorac Vasc Anesth. (2005) 19:150–4. 10.1053/j.jvca.2005.01.022 15868519

[26] Sukernik MR Bennett-Guerrero E. The incidental finding of a patent foramen ovale during cardiac surgery: should it always be repaired? A core review. Anesth Analg. (2007) 105:602–10. 10.1213/01.ane.0000278735.06194.0c 17717210

[27] Lo TT Jarral OA Shipolini AR McCormack DJ. Should a patent foramen ovale found incidentally during isolated coronary surgery be closed? Interact Cardiovasc Thorac Surg. (2011) 12:794–8. 10.1510/icvts.2011.265835 21345819

[28] Krasuski RA Hart SA Allen D Qureshi A Pettersson G Houghtaling PL. Prevalence and repair of intraoperatively diagnosed patent foramen ovale and association with perioperative outcomes and long-term survival. Jama. (2009) 302:290–7. 10.1001/jama.2009.1012 19602688

[29] Goel SS Tuzcu EM Shishehbor MH de Oliveira EI Borek PP Krasuski RA. Morphology of the patent foramen ovale in asymptomatic versus symptomatic (stroke or transient ischemic attack) patients. Am J Cardiol. (2009) 103:124–9. 10.1016/j.amjcard.2008.08.036 19101242

[30] Bucerius J Gummert JF Borger MA Walther T Doll N Onnasch JF. Stroke after cardiac surgery: a risk factor analysis of 16,184 consecutive adult patients. Ann Thorac Surg. (2003) 75:472–8. 10.1016/S0003-4975(02)04370-9 12607656

[31] Hogue CW Murphy SF Schechtman KB Dávila-Román VG. Risk factors for early or delayed stroke after cardiac surgery. Circulation. (1999) 100:642–7. 10.1161/01.CIR.100.6.642 10441102

[32] Sultan I Bianco V Kilic A Jovin T Jadhav A Jankowitz B. Predictors and outcomes of ischemic stroke after cardiac surgery. Ann Thorac Surg. (2020) 110:448–56. 10.1016/j.athoracsur.2020.02.025 32199830

[33] Gaudino M Rahouma M Di Mauro M Yanagawa B Abouarab A Demetres M. Early versus delayed stroke after cardiac surgery: a systematic review and meta-analysis. J Am Heart Assoc. (2019) 8:e012447. 10.1161/JAHA.119.012447 31215306 PMC6662344

[34] Djaiani G Phillips-Bute B Podgoreanu M Messier RH Mathew JP Clements F. The association of patent foramen ovale and atrial fibrillation after coronary artery bypass graft surgery. Anesth Analg. (2004) 98:585-9. 10.1213/01.ANE.0000099721.67426.DE 14980902

[35] Kusunose K Yamada H Todoroki T Nishio S Niki T Yamaguchi K. Platypnea-orthodeoxia syndrome associated with patent foramen ovale and aortic ectasia. Echocardiography. (2009) 26:114–7. 10.1111/j.1540-8175.2008.00780.x 19017323

[36] Godart F Rey C Prat A Vincentelli A Chmaït A Francart C. Atrial right-to-left shunting causing severe hypoxaemia despite normal right-sided pressures. Report of 11 consecutive cases corrected by percutaneous closure. Eur Heart J. (2000) 21:483–9. 10.1053/euhj.1999.1944 10681489

[37] Mojadidi MK Zaman MO Elgendy IY Mahmoud AN Patel NK Agarwal N. Cryptogenic Stroke and Patent Foramen Ovale. J Am Coll Cardiol. (2018) 71:1035–43. 10.1016/j.jacc.2017.12.059 29495983

