# Supplementary material for: Risk factors and complications associated with intra-operative or post-operative identification of a PFO in cardiac surgery patients: A cohort study
Source: Front Neurol. 2023 Jan 10;13:1057479. doi: 10.3389/fneur.2022.1057479 (PMC9871930; doi:10.3389/fneur.2022.1057479)
Supplement: Supplementary file 1 [file Table_1.DOCX]

**Supplemental Table 1. Predictors of PFO reopening**

|  | Univariate  OR 95%CI | p | Multivariate  OR 95%CI | | p |
| --- | --- | --- | --- | --- | --- |
| Baseline characteristics |  |  |  | |  |
| Age per years | 0.98 (0.97-0.995) | 0.004 | - | |  |
| Men | 0.87 (0.65-1.2) | 0.34 |  | |  |
| Smoker status | 0.95 (0.72-1.2) | 0.75 |  | |  |
| Hypertension | 0.81(0.63-1.06) | 0.13 |  | |  |
| Diabetes | 0.47(0.32-0.69) | 0.001 | - |  | |
| Body mass index (kg/m^2^) | 0.95 (0.93-0.98) | 0.003 | - | |  |
| Chronic obstructive pulmonary disease | 1.4 (0.83-2.2) | 0.21 |  | |  |
| Left ventricular ejection function ≥ 50% | 1.4 (0.94-2.0) | 0.09 | - | |  |
| Left ventricular ejection function 31-49% | 0.64(0.41-0.99) | 0.04 | - | |  |
| Left ventricular ejection function ≤ 30% | 1.2(0.56-2.6) | 0.59 |  | |  |
| Peripherical arterial disease | 0.95(0.62- 1.4) | 0.81 |  | |  |
| Pulmonary artery systolic pressure >35mmHg | 1.5 (1.1-2.0) | 0.004 | - | |  |
| Pulmonary artery systolic pressure >55mmHg | 1.8 (1.1-3.1) | 0.017 | - | |  |
| Previous atrial fibrillation | 2.7 (1.9-3.8) | 0.0001 | 1.6 (1.1-2.3) | | 0.01 |
| Per-operative characteristics |  |  |  | |  |
| Coronary bypass grafting | 0.33 (0.25-0.43) | 0.001 | 0.32 (0.21-0.50) | | 0.001 |
| Mitral valve surgery | 4.4 (3.4-5.8) | 0.001 | 1.2 (0.8-1.8) | | 0.41 |
| Aortic valve surgery | 0.36 (0.25-0.52) | 0.001 | 0.25(0.16-0.38) | | 0.0001 |
| Ascending aorta surgery | 1.45(0.94-2.2) | 0.10 | 1.7 (1.01-2.8) | | 0.048 |
| Tricuspid valve surgery | 9.7 (7.0-13.4) | 0.001 | 3.7(2.6-5.5) | | 0.0001 |
| Urgent surgery | 0.88 ( 0.48- 1.6) | 0.66 |  | |  |
| Redux | 0.73(0.45-1.2) | 0.19 |  | |  |
|  |  |  |  | |  |

|  | Univariate  OR 95%CI | p | Multivariate  OR 95%CI | | p |
| --- | --- | --- | --- | --- | --- |
| Baseline characteristics |  |  |  | |  |
| Age (per years) | 1.04 (1.02-1.05) | 0.0001 | 1.02 (1.01-1.04) | | 0.0001 |
| Men | 1.5(1.8-2.0) | 0.001 | 1.2 (0.9-1.5) | | 0.26 |
| Smoker status | 0.78 (0.58-1.05) | 0.10 |  | |  |
| Hypertension | 1.15(0.89-1.5) | 0.27 |  | |  |
| Diabetes | 0.72 (0.52-0.99) | 0.04 | - |  | |
| Body mass index (per point) | 0.95(0.93-0.98) | 0.005 | - | |  |
| Chronic obstructive pulmonary disease | 0.80 (0.44-1.5) | 0.50 |  | |  |
| Left ventricular ejection function ≥ 50% | 0.91(0.66-1.2) | 0.57 |  | |  |
| Left ventricular ejection function 31-49% | 1.1(0.78-1.6) | 0.55 |  | |  |
| Left ventricular ejection function ≤ 30% | 0.98(0.43-2.2) | 0.98 |  | |  |
| Peripherical arterial disease | 1.2 (0.8-1.7) | 0.4 |  | |  |
| Elevated pulmonary artery systolic pressure above 35mmHg | 1.6(1.2-2.0) | 0.001 | 1.1(0.9-1.5) | | 0.38 |
| Previous atrial fibrillation | 1.7(1.2-2.4) | 0.007 | - | |  |
| Post operative atrial fibrillation | 1.3(1.01-1.8) | 0.05 | 0.8(0.55-1.15) | | 0.23 |
| PFO | 2.0 (1.05-3.8) | 0.04 | - | |  |
| Post operative disclosure of PFO | 3.5(1.6-7.7) | 0.001 | 3.5(1.6-7.8) | | 0.002 |
| Per operative closure of PFO | 0.97(0.3-3.0) | 0.97 |  | |  |
| Per-operative characteristics |  |  |  | |  |
| Coronary bypass grafting | 0.49(0.38-0.63) | 0.0001 | - | |  |
| Mitral valve surgery | 2.1 (1.6-2.8) | 0.0001 | 2.4(1.8-3.3) | | 0.0001 |
| Aortic valve surgery | 1.5 (1.2-1.9) | 0.002 | - | |  |
| Ascending aorta surgery | 2.4(1.6-3.4) | 0.0001 | 3.1 (2.1-4.5) | | 0.0001 |
| Tricuspid valve surgery | 1.7(0.99-2.9) | 0.055 | - | |  |

**Supplemental Table 2. Predictors of cerebral ischemic event**

**Supplemental Table 3. Outcomes of patients with reopening PFO depending on whether or not they are closed.**

|  | Per or post operative PFO closure (n=151) | No PFO closure (n=82) | p |
| --- | --- | --- | --- |
| Pneumonia | 18 (11.9) | 24 (29.3) | 0.002 |
| Reintubation | 8(5.2) | 11 (13.4) | 0.03 |
| Duration of mechanical  Ventilation(hours) | 6 [4-10] | 4.5[4-13] | 0.79 |
| Cerebral ischemic event | 4 (2.6) | 6 (7.3) | 0.17 |
| Comitial event | 3 (2.0) | 2 (2.4) | 0.81 |
| Diaphragmatic paralysis | 5 (3.3) | 10 (12.2) | 0.01 |
| New onset of postoperative  atrial fibrillation | 68 (45.0) | 19(23.0) | 0.001 |
| Vasopressors use | 42 (27.8) | 13 (15.8) | 0.05 |
| Mediastinitis | 1 (0.7) | 1 (1.2) | 0.54 |
| Length of stay in ICU | 4[3-5] | 5.5 [4-8] | <0.001 |
| Length of stay in hospital.  days | 14[12-18] | 16 [13-22] | 0.047 |
| In-hospital mortality | 5 (3.3) | 7 (8.5) | 0.08 |

**Figure Legend**

**Supplemental Figure 1. Flow chart**
