# Supplementary figures and images for: Risk factors and complications associated with intra-operative or post-operative identification of a PFO in cardiac surgery patients: A cohort study
Source: Front Neurol. 2023 Jan 10;13:1057479. doi: 10.3389/fneur.2022.1057479 (PMC9871930; doi:10.3389/fneur.2022.1057479)

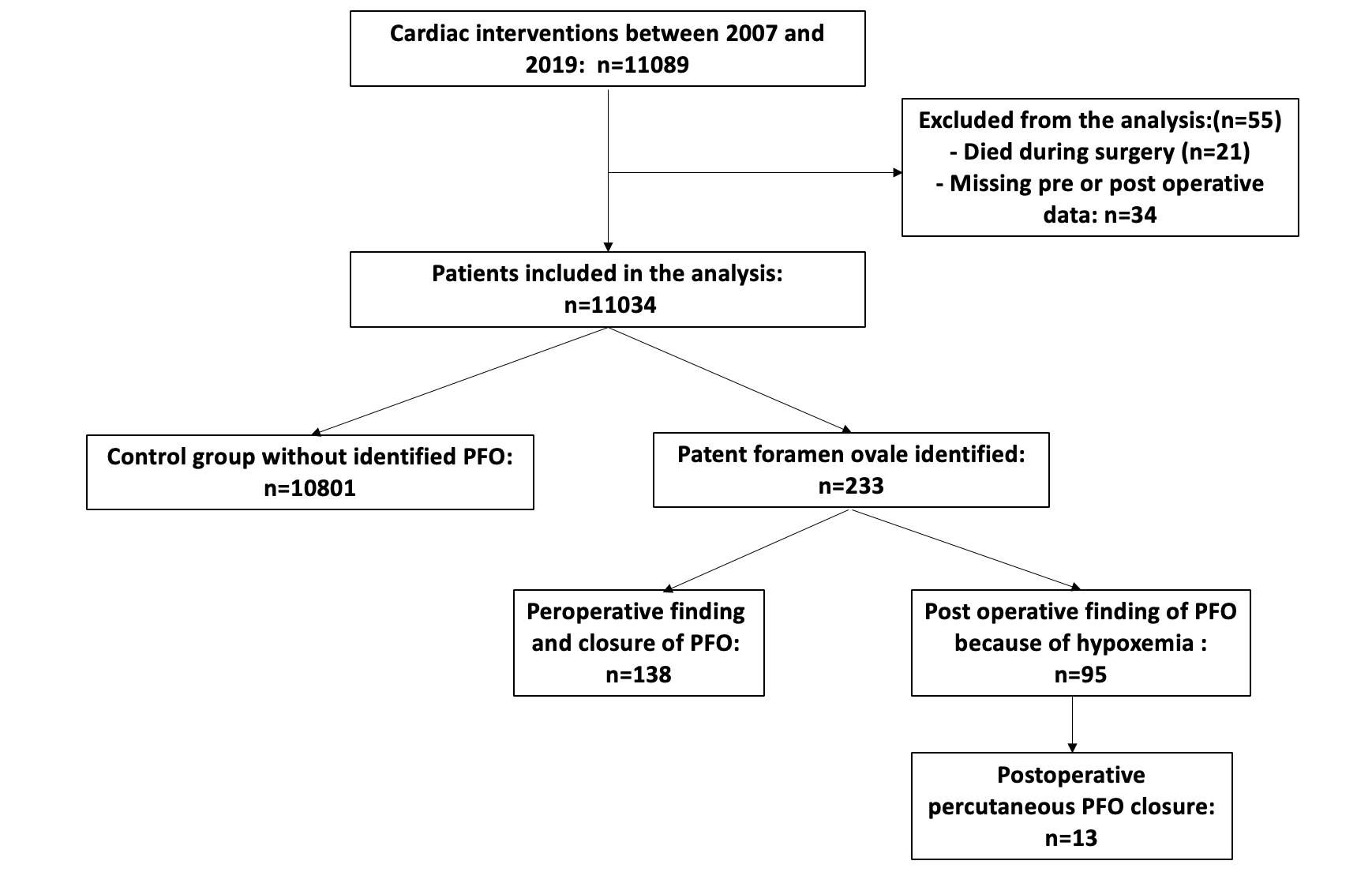

Supplement: Supplementary file 2 [file Image_1.PNG]
